# Beyond *mecA*: a two-tiered mechanism and regulatory rewiring drive high-level ceftaroline resistance in clinical MRSA

**DOI:** 10.1128/aac.00586-25

**Published:** 2026-01-07

**Authors:** Melissa Sassman, Michele Karolak, Margeurite Dallaire, Claire E. Schaffer, Carlos Gartner, Calvin Vary, Roberto R. Rosato, Adriana E. Rosato

**Affiliations:** 1 Center for Molecular Medicine, MaineHealth Institute for Research 138817 , Scarborough, Maine, USA; 2 Tufts University School of Medicine 12261, Boston, Massachusetts, USA; 3 Graduate School of Biomedical Science and Engineering, University of Maine 6250, Orono, Maine, USA; The Peter Doherty Institute for Infection and Immunity, Melbourne, Victoria, Australia

**Keywords:** MRSA, ceftaroline, resistance mechanisms, *rpoB*, cell wall remodeling

## Abstract

High-level resistance to ceftaroline, a fifth-generation β-lactam critical for treating methicillin-resistant *Staphylococcus aureus* (MRSA), is an emerging threat to global health. While resistance is traditionally attributed to *mecA*-mediated expression of PBP2a, our study reveals a previously unrecognized mechanism. We show that high-level resistance to ceftaroline can arise independently of ceftaroline exposure through a collateral pathway triggered by carbapenems typically used to treat Gram-negative infections. Our findings reveal a two-tiered adaptive process. First, meropenem selects non-synonymous mutations in *rpoB*, a core transcriptional regulator, which primes resistance by reprogramming gene expression. These changes consistently co-occur with a key substitution in *pbp1* (H499R), an essential protein for cell division, and specific *mecA* variants (Y446H, E447K) following ceftaroline exposure. Second, resistance is stabilized through regulatory and signaling adaptations, with elevated basal levels of the oxidative stress regulator Spx and its adaptor protein TrfA supporting the altered cellular state. Proteomic and biophysical studies revealed direct binding of TrfA to GdpP, the phosphodiesterase for cyclic-di-AMP, linking this regulatory circuit to elevated c-di-AMP levels and resistance maintenance. Our findings challenge the assumption that ceftaroline resistance is driven solely by PBP2a alterations and reveal how collateral resistance pathways can be activated by broad-spectrum antibiotic use. This study highlights the evolutionary capacity of MRSA to circumvent antibiotic pressure and underscores the need for improved antimicrobial stewardship.

## INTRODUCTION

Antimicrobial resistance (AMR) poses a growing threat to global health, contributing to an estimated 4.95 million deaths in 2019 (1). Methicillin-resistant *Staphylococcus aureus* (MRSA) is among the most persistent and clinically challenging pathogens, causing infections ranging from bacteremia and pneumonia to osteomyelitis and endocarditis in both healthcare and community settings. MRSA’s ability to evade multiple antibiotic classes, particularly β-lactams, has made it a priority pathogen for new treatment strategies.

Ceftaroline, a fifth-generation cephalosporin, represents a critical advance in β-lactam therapy against MRSA due to its high-affinity binding penicillin-binding protein 2a (PBP2a), the product of the *mecA* resistance gene. This mechanism restores β-lactam activity by targeting a transpeptidase that other β-lactams cannot inhibit. However, resistance to ceftaroline is increasingly reported, with clinical isolates exhibiting rates between 3% and 15% (2–6). Surprisingly, clinical cases of CPT-resistant MRSA infections have been observed without prior CPT exposure (7–9), indicating that resistance is not exclusively driven by direct selection pressure but may arise through alternative adaptive mechanisms (2–6). CPT resistance in MRSA is typically attributed to *mecA*-mediated alterations in PBP2a, leading to low-level resistance (MICs 2–4 µg/mL). Our clinical observations and comparative genomics point to a reproducible multistep path to high- level CPT resistance. In one case, an MRSA strain (SA5007) exhibited high-level resistance (minimum inhibitory concentration, MIC ≥ 32 µg/m L) after only one day of CPT exposure in the clinic, implying the presence of potentiating factors. Our previously published phylogenetic analysis of 162 MRSA strains revealed two distinct clades: one susceptible to both meropenem and CPT, and another resistant to both. Whole-genome sequencing of the resistant clade revealed key mutations driving resistance: *rpoB* mutations (H481N, S464P, Q468L, or D471A), which could serve as resistance potentiators; *pbp1* H499R mutation at the PBP1 transpeptidase domain; and *mecA* mutations (E239K, Y446N, E447K), which enhance CPT resistance, suggesting a stepwise adaptive mechanism.

To complement these clinical observations, *in vitro* evolution experiments were conducted using representative strains from the clade of ceftaroline- and carbapenem-susceptible isolates. These experiments confirmed that carbapenem (e.g., meropenem or imipenem) pre-treatment followed by ceftaroline (CPT) exposure reliably induces high-level resistance. Early exposure to meropenem or imipenem selected for mutations in *rpoB*, and subsequent CPT exposure (by day 15) led to additional mutations, including *mecA* (E447K) and *pbp1* (H499R). By day 25, the evolved strain exhibited a genetic profile nearly identical to the highly resistant clinical isolate SA5007 (CPT MIC: 32 µg/mL), carrying *mecA* E239K, Y446N, and E447K mutations. In contrast, repeated CPT exposure alone did not produce this resistance phenotype (6). Mutations in *rpoB* and *rpoC*, encoding the β and β′ subunits of RNA polymerase, are well-established contributors to altered levels of antibiotic resistance (7).

The high level of ceftaroline resistance in clinical MRSA strains appears to follow a reproducible multistep process, in which *rpoB* mutations consistently appear first, followed by subsequent changes associated with cell wall synthesis and energy metabolism. We primarily found that *rpoB* mutations were associated with cystic fibrosis MRSA clinical isolates, but recently, we observed the extension of its role to MRSA clinical strains obtained from cancer patients. Both patient populations are well-documented to receive repeated courses of carbapenems. In cystic fibrosis, meropenem is routinely used to treat pulmonary exacerbations, often at high doses or as prolonged infusions (8). Likewise, in oncology patients with febrile neutropenia, meropenem is widely prescribed as empiric broad-spectrum therapy, with reports documenting its use in the majority of such episodes (9, 10).

A high level of ceftaroline resistance is also associated with PBP1 alterations. In *S. aureus*, the essential roles of penicillin-binding proteins—(PBPs) 1 and 2—in peptidoglycan synthesis are critical for bacterial cell wall integrity. PBP1, with exclusive transpeptidase (TP) activity, and PBP2, bearing both TP and transglycosylase (TG) activities, are indispensable for septal and peripheral PG formation (11–13). PBP2 is crucial for peptidoglycan synthesis throughout the cell cycle. However, in methicillin-resistant *S. aureus* (MRSA), PBP2’s TP function can be compensated by PBP2a, though PBP2a cannot substitute for PBP1, whose absence proves lethal. PBP1, a transpeptidase that works with its cognate partner transglycosylase FtsW, serves a vital role in the life cycle of *S. aureus,* overseeing cell division at the septum, marked by its distinct dense concentric ring peptidoglycan architecture (12, 14).

In this study, we demonstrate that high- level CPT resistance emerges through a broader, multistep process involving *rpoB* mutations that modulate global transcription, cell wall remodeling through PBP1 substitutions, and signal transduction shifts within the TrfA–GdpP –cdiAMP axis. Specifically, we examine the role of *rpoB* in potentiating CPT resistance and investigate how regulatory circuits—Spx, its adaptor TrfA, and cdiAMP signaling—contribute to the stabilization of high-level resistance.

These findings integrate with our earlier results and challenge the *mecA*-centric paradigm, revealing previously unrecognized collateral resistance. This integrated perspective has implications for antimicrobial stewardship, resistance surveillance, and therapeutic development.

## RESULTS

### *rpoB* mutations may act as potentiators of high-level ceftaroline resistance

A growing body of evidence links *rpoB* mutations, often but not always, to conferring resistance to rifampicin and changes in cellular responses, strongly suggesting changes in the properties of the RNA polymerase (15–17). These changes include the activation of cryptic metabolism, alterations in transcriptomic profiles, and associations with drug resistance, particularly to β-lactams. The observation that *rpoB* is primarily associated with ceftaroline resistance led us to hypothesize that *rpoB* mutations may contribute as potentiators to sustain the survival of MRSA in the presence of ceftaroline.

**Fig 1:**
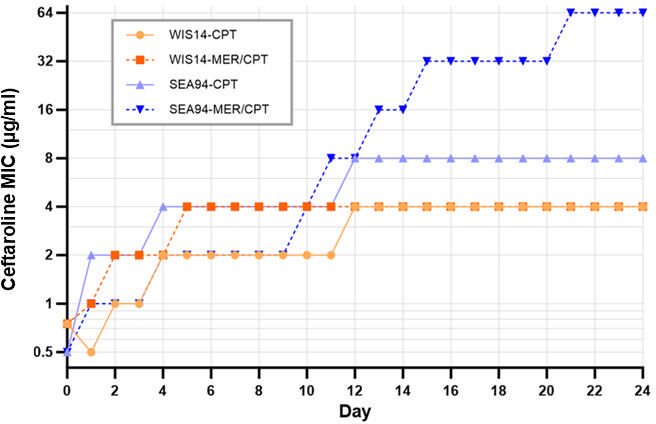
Evolutionary selection of ceftaroline resistance. Clinical MRSA strains WIS_14 and SEA_94 were subjected to two experimental conditions, direct exposure to ceftaroline (CPT) or pre-exposure to meropenem followed by ceftaroline selection. Strains were cultured in progressively increasing concentrations of CPT over 24 days. Minimum inhibitory concentrations (MICs) were monitored daily to evaluate phenotypic resistance development (Table 2).

To test this hypothesis, we performed evolutionary experiments using two MRSA strains, each exposed for 24 days to ceftaroline, meropenem, or meropenem followed by ceftaroline, or left untreated as a control. Daily phenotypic monitoring and whole-genome sequencing revealed that high-level ceftaroline resistance (MIC ≥32 µg/mL) only emerged in strains harboring non-synonymous *rpoB* mutations (S454P) (e.g., SEA-94 strain, Fig. 1). Strains lacking *rpoB* mutations or carrying only synonymous *rpoB* variants failed to develop high-level ceftaroline resistance (WIS14; Fig. 1). Whole-genome sequencing of these lineages revealed the emergence of several key mutations, notably the H499R substitution in *pbpA* (*pbp1*, encoding PBP1), exclusively in high-level resistant strains such as SEA94. Strains such as WIS14, which reached a lower level of ceftaroline resistance, harbored only synonymous mutations in *pbp1* (Table 1). These observations reinforce the connection between these mutations and elevated MICs (Table 2). These results highlight the critical role of *rpoB* mutations in driving high-level ceftaroline resistance and demonstrate a marked divergence in the evolution of resistance between MRSA strains.

**TABLE 1:** Mutational profile of *S. aureus* strains WIS14 and SEA94 after exposure to ceftaroline (CPT), meropenem (MER), or their combination (MER–CPT)^*a*^

Ref ID	Ref Pos	Gene Name	WIS_14 ⁄ SEA_94	CPT	MER	MER-CPT
WIS14						
NC_002745	580228	*rpoB*	T > C	p.(=)	p.(=)	
NC_002745	581509	*rpoB*	C > T	p.(=)	p.(=)	
NC_002745	581593	*rpoB*	A > T	p.(=)	p.(=)	
NC_002745	581632	*rpoB*	A > G	p.(=)	p.(=)	
NC_002745	581692	*rpoB*	A > T	p.(=)	p.(=)	
NC_002745	46254	*mecA*		C > T (E262K)	C > T (E262K)	C > T (E262K)
NC_002745	1159457	*pbpA*	T > Gp.(=)	T > Gp.(=)	T > Gp.(=)	T > Gp.(=)
SEA_94						
NC_002745	581043	*rpoB*			S454P	
NC_002745	45699	*mecA*		C > T (E447K)		C > T
NC_002745	45702	*mecA*		A > G (Y446H)		A > G
NC_002745	46963	*mecA*	G > T	G > T [p.(=)]	G > T [p.(=)]	G > T [p.(=)]
NC_002745	1159549	*pbpA*		A > G (H499R)		A > G (H499R)

^*a*^ Nucleotide substitutions and corresponding amino acid changes were identified relative to the *S. aureus* N315 (GenBank NC_002745). Synonymous mutations are shown as p.(=); non-synonymous as specific amino acid changes.

**TABLE 2:** Stepwise selection of high-level ceftaroline resistance in CF-MRSA clinical strains and associated genetic adaptations^*a*^

	D7		D14		D24	
STRAINS	MER	CPT	MER	CPT	MER	CPT
SEA 94	0.75	0.5	0.75	0.5	0.75	0.5
SEA 94-MER	>32	0.5	>32	2	>32	1
SEA 94-CPT	>32	1.5	>32	1.5	>32	>8
SEA 94-MER/CPT	>32	0.75	>32	3	>32	>32
WIS 14	0.38	0.5	0.75	0.5	0.75	0.5
WIS 14-MER	128	1	128	2	128	2
WIS 14-CPT	128	2	128	2	>32	2
WIS 14-MER/CPT	128	4	128	4	>32	4

^*a*^ (A) Phenotypic resistance profiles of SEA 94 and WIS 14 strains during exposure to ceftaroline (CPT) and/or meropenem (MER) over 24 days. Minimal inhibitory concentrations (MICs, µg/mL) of MER and CPT were determined at days 7 (D7), 14 (D14), and 24 (D24) for parental strains and derivative populations selected under: Meropenem (MER), Ceftaroline (CPT), sequential MER followed by CPT (MER/CPT).

To assess the functional role of *rpoB* mutations in potentiating ceftaroline resistance, we cloned a mutated *rpoB* allele (D471G, S486L) from strain WIS7-185 into the wild-type WIS-7, generating the WIS-7(*rpoB^+^*) strain. The construct was exposed to imipenem and/or meropenem (8 µg/mL) followed by progressive ceftaroline selection up to 128 µg/mL over 7 days, resulting in ceftaroline MICs of 32 µg/mL—compared to 4 µg/mL for the wild-type WIS-7 strain under identical conditions. These findings underscore that *rpoB* mutations confer a selective advantage by acting as potentiators of ceftaroline resistance (Table 3)

**TABLE 3:** Phenotypic analysis of *rpoB* mutations in potentiating ceftaroline resistance in MRSA^*a*^

	D0		D2		D7	
STRAINS	MER	CPT	MER	CPT	MER	CPT
WIS 7 (*rpoB* WT)	0.75	0.5	0.75	0.5	0.75	0.75
WIS 7 - MER (*rpoB* WT)	16	0.5	16	0.75	16	0.75
WIS 7 – MER/CPT (*rpoB* WT)	>32	1	>32	4	>32	4
WIS 7 (*rpoB+*)	0.75	0.5	0.75	2	0.75	2
WIS 7 (*rpoB+*) MER	≥128	2	≥128	2	≥128	4
WIS 7 (*rpoB+*) MER/CPT	≥128	2	≥128	16	≥128	32

^*a*^ Phenotypic resistance profiles of parental WIS7 strain and its isogenic strain containing cloned *rpoB* mutations. phenotypic resistance profiles of WIS 7 (*mecA+*, positive) and WIS 7 (*mecA+, rpoB+*) strains during exposure to imipenem IMP and/or meropenem (MER) over 7 days and ceftaroline.

Consistent with these results, serial passage experiments (Table 4) with additional strains showed that those harboring *rpoB* mutations (P1063S, D471G, P475T, S464P) developed markedly higher ceftaroline MICs over 25 days compared to wild-type strains. While wild-type isolates such as Wis-67 and MTH-84 showed only modest increases (1–8 µg/mL), mutant strains such as SA149 (*rpoB* P1063S) and SA93 (*rpoB* P475T) reached MICs of 64–256 µg/mL, particularly under combined meropenem/ceftaroline exposure. These data confirm that *rpoB* mutations facilitate the evolution of high-level ceftaroline resistance during β-lactam selection and support a mechanistic link between *rpoB* alterations and carbapenem-driven adaptation in MRSA.

**TABLE 4:** Evolution of MIC over serial passages in the strains harboring either wild-type (WT) or mutated *rpoB* and the corresponding MIC to ceftaroline

		Day 0			Day 7			Day 15			Day 25		
Strains	*rpoB*	MER	CPT	CPT/MER	MER	CPT	CPT/MER	MER	CPT	CPT/MER	MER	CPT	CPT/MER
WIS-67	WT	0.5	1	0.5	3	2	3	3	3	4	8	8	8
MTH-84	WT	0.5	0.5	0.5	4	1	4	6	2	4	6	6	6
MTH-2	WT	0.38	0.75	0.5	3	1.5	2	3	2	1.5	4	6	4
SA149	P1063S	0.5	0.5	0.5	128	2	2	128	8	8	256	8	64
SA113	D471G	0.5	1	0.5	32	1	3	3	3	4	8	8	32
SA93	P475T	0.5	0.75	0.5	16	1	3	32	4	12	32	6	32
MTH 87	S464P	0.38	0.75	0.5	2	3	2	1.5	4	4	4	8	32

To assess the clinical relevance of *rpoB* mutations in additional patient populations potentially predisposed to ceftaroline resistance because of related carbapenem indications (e.g., cancer patients), we performed whole-genome sequencing on additional MRSA strains (Table 5). *rpoB* mutations were detected in 30% of those isolates. Taken together, these findings provide mechanistic support for our evolutionary and clinical observations, underscoring the role of *rpoB* mutations in driving high-level ceftaroline resistance. Moreover, the prevalence of *rpoB* mutations appears to extend to other patient populations, including non-cystic fibrosis individuals.

**TABLE 5:** Association of *rpoB* variants with meropenem (MER), imipenem (IMP), ceftaroline (CPT), and oxacillin (OXA) MICs in clinical MRSA isolates from diverse infections

Type of strains	Strains	(*rpoB+*) mutations	MER	IMP	CPT	OXA
CF	SEA_3	H481N	4	8	32	>256
CF	SEA_122	H481N	16	32	16	>256
CF	SEA_95	D471G	16	32	32	>256
NON-CF strains						
Bacteremia	MTH_50	H481Y	6	6	12	>256
Pneumonia	MTH_76	A455T, S464P	4	12	32	>256
Bacteremia	SA196	A477D, D471G	8	8	16	>256
Bacteremia	SA413	A477D, D471G	16	8	32	>256

### Role of mutated (H499R) PBP1 in high-level ceftaroline resistance

In *S. aureus*, the essential roles of penicillin-binding proteins—(PBPs) 1 and 2— in peptidoglycan synthesis are critical for bacterial cell wall integrity. PBP1, with exclusive transpeptidase (TP) activity, and PBP2, bearing both TP and transglycosylase (TG) activities, are indispensable for septal and peripheral PG formation (11–13). Our initial findings from clinical and *in vitro* evolved CPT-resistant strains indicate that transition from CPT-susceptible is favored from the pre-exposure to carbapenems. Carbapenems (e.g., meropenem) can allosterically bind PBP2a, influencing CPT susceptibility (15).

**Fig 2:**
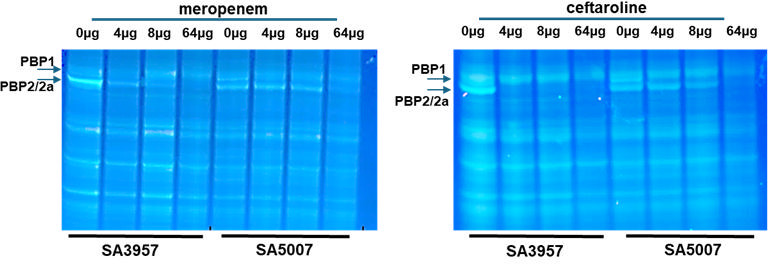
Analysis of PBP levels in membrane preparations. Competitive binding assays with Bocillin FL were conducted on membrane proteins obtained from ceftaroline-susceptible SA3957 and ceftaroline-resistant SA5007 cells pre-incubated with varying concentrations of meropenem or ceftaroline at the concentrations indicated in the figure. Equal amounts (20 µg) of Bocillin FL-labeled membrane proteins were separated by 4–12% SDS-PAGE. Arrows indicate fluorescently labeled PBP1 and PBP2/2a.

Carbapenems, such as meropenem and imipenem, show high affinity for PBP1, and in *S. aureus*, inhibition of PBP1 by carbapenems can disrupt cell wall synthesis and division. We have found that the (H499R) PBP1 mutation is associated with high-level ceftaroline resistance (6). To ascertain whether the H499R mutation located at the TP site in PBP1 impacts its binding affinity to meropenem, competitive binding assays with Bocillin FL were conducted on membrane proteins pre-incubated with varying concentrations of meropenem (Fig. 2). Reduced antibiotic binding (i.e., increased Bocillin FL affinity) was observed in SA5007 cells grown in the presence of meropenem, suggesting that the H499R mutation at the active site results in diminished binding affinity for meropenem. Similarly, in the meropenem-resistant but ceftaroline-susceptible strain SA3957, there was a less pronounced reduction in binding affinity. Thus, these findings indicate that the H499R mutation may detrimentally affect PBP1’s binding capacity to β-lactams. In SA3957 treated with varying concentrations of ceftaroline, increased binding to PBP2/PBP2a was observed compared to ceftaroline-resistant SA5007, which exhibited no binding except at a concentration of 64 µg/mL (above the MICs of 32 µg/mL). This paradoxical expression pattern may reflect a compensatory regulatory mechanism triggered by impaired protein function or altered localization.

**Fig 3:**
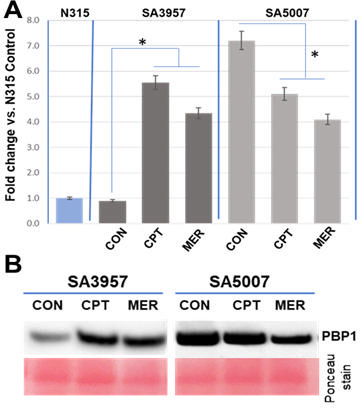
Analysis of PBP1 mRNA and protein levels. PBP1 mRNA (**A**) and protein (**B**) levels were analyzed in ceftaroline-susceptible SA3957 and ceftaroline-resistant SA5007 cells treated with ceftaroline or meropenem (2/32 µg/mL and 8/8 µg/mL, respectively). *pbpA* gene expression was determined by qRT-PCR using RNA from treated cell lysates; relative values vs SA-N315 = 1 compared; PBP1 protein levels were evaluated by Western blot of cell membrane samples, detected with an anti-PBP1 antibody. (*) Significantly different (ANOVA), *P* < 0.01; loading control: Ponceau staining.

To determine whether the H499R mutation also impacts the expression dynamics of PBP1, we analyzed PBP1 expression by Western blot and RT-PCR in both susceptible parental SA3957 and resistant SA5007 strains, ± antibiotic treatment. Increased PBP1 mRNA and protein levels were observed in SA3957 upon exposure to meropenem (32 µg/mL) and ceftaroline (2 µg/mL; Fig. 3). In contrast, expression of PBP1, which was constitutively higher in SA5007 at baseline levels, decreased when exposed to antibiotics (meropenem/ceftaroline: 8 µg/mL; Fig. 3). These findings collectively suggest that the H499R mutation in PBP1 contributes to high-level ceftaroline resistance by both impairing β-lactam binding and altering the expression dynamics of PBP1. In the resistant SA5007 strain, PBP1 is constitutively overexpressed and downregulated upon antibiotic exposure, in contrast to the inducible expression observed in the susceptible SA3957. This regulatory shift, combined with decreased antibiotic binding, underscores the critical role of mutated PBP1 in mediating ceftaroline resistance.

### Levels of Spx and TrfA are associated with high-level of ceftaroline resistance

Previous research from Renzoni’s group provided compelling evidence that TrfA is an integral component of the cell wall stress regulon and is linked to intermediate resistance to glycopeptides, such as teicoplanin, vancomycin, and oxacillin (16). Specifically, these cell wall inhibitors selectively induced *trfA* mRNA and TrfA protein levels, a response not elicited by other antibiotic types. The Spx-TrfA regulatory pathway is involved in bacterial stress responses including oxidative stress and antibiotic exposure (17–19). TrfA is predicted to be an adaptor homologous to MecA of *B. subtilis* (note: MecA of *B. subtilis* has no functional relationship with PBP2a encoded by *mecA* that confers the MRSA phenotype). In a previous study, TrfA, together with ClpCP (a chaperone/protease pair), was shown to degrade proteins with a *ssrA*-tag (20), a degradation motif for the bacterial proteasome. The redox-sensitive regulator Spx controls the temporal transcription of *trfA* and is degraded by YjbH-ClpXP (21), while it self-represses by binding to its own promoter. YjbH, only active in its soluble non-aggregated forms, functions as a chaperone. This has been identified in several studies examining antibiotic-resistant strains, including those resistant to β-lactams, further linking YjbH/Spx to antibiotic resistance (21–23).

**Fig 4:**
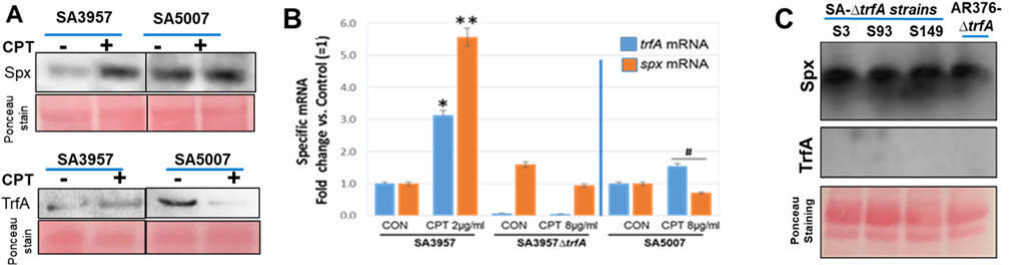
Levels of Spx and TrfA are associated with high-level ceftaroline resistance. (**A**) Western blot analysis of Spx and TrfA protein levels in MRSA ceftaroline-susceptible (SA3957) and -resistant (SA5007) clinical strains treated with ceftaroline (CPT), or meropenem (MEM) at the indicated concentrations (µg/mL). Ponceau staining was used as a loading control. (**B**) Quantitative RT-PCR analysis of target genes (spx, trfA) involved in oxidative stress response pathways following antibiotic treatment in ceftaroline-susceptible (SA3957; ** p< 0.001) and -resistant strains (SA5007, SA3957ΔtrfA; * p< 0.05–0.01). Expression level is presented as fold change (mean ± SD, n = 3) relative to untreated SA3957 control (= 1). (**C**), Western blot analysis of Spx and TrfA expression in trfA-null SA3, S39, S149, and AR376 (control) strains. Ponceau staining was used as a loading control.

Consistent with the known mechanisms described of the Spx/TrfA pathway, we observed increased protein levels of both Spx and TrfA in ceftaroline-susceptible SA3957 following exposure to ceftaroline (Fig. 4A). However, the ceftaroline-resistant SA5007 strain displayed increased protein levels of Spx at the basal level and under ceftaroline exposure, suggesting Spx accumulation. TrfA protein levels were significantly decreased upon SA5007 CPT treatment compared to SA3097 increased levels.

Transcriptional analysis of *trfA* and *spx* revealed increased levels in ceftaroline-susceptible SA3957 cells treated with ceftaroline *vs*. untreated cells; by contrast, minimal changes in *trfA* and *spx* gene expression were observed in the ceftaroline-resistant strain SA5007 (Fig. 4B).

To investigate the functional significance of TrfA in ceftaroline resistance, we generated an isogenic *trfA*-null mutant in the ceftaroline-susceptible SA3957 background strain (SA3957*ΔtrfA*). The inactivation of *trfA* was achieved by allelic replacement integrating the *tetK* gene (AR376 *ΔtrfA::tetK*) (19). Phenotypic analysis of the SA3957*ΔtrfA* null mutant revealed a significant increase in resistance to ceftaroline, with MICs reaching 32 µg/mL from the original 2 µg/mL. Complementation of SA3957*ΔtrfA* by expressing *trfA* on a plasmid restored ceftaroline sensitivity (MIC: 1 µg/mL; Table 7). Interestingly, the SA3957*ΔtrfA* mutant had decreased *spx* gene expression under ceftaroline exposure. Western blot analysis of Spx and TrfA showed clear absence of TrfA protein under either condition, accompanied by sustained high levels of Spx (Fig. 4C). Similar results were obtained with the control *trfA*-null mutant AR376-*ΔtrfA* strain (19). Together, these findings indicate a distinct pattern wherein impairment in the Spx/TrfA pathway contributes to the emergence of ceftaroline resistance.

**Fig 5:**
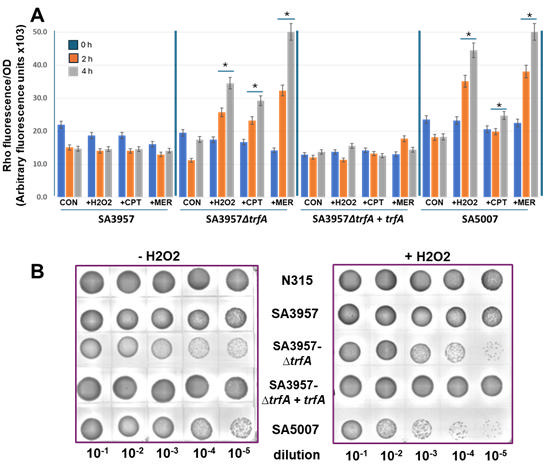
(**A**) Levels of reactive oxygen species (ROS) were measured in cells treated with DHR123. Fluorescence was normalized to cell density (OD-600nm). Hydrogen peroxide (H2O2; 1 mM) was used as a positive control. Treatments included SA3957 and SA3957*∆trfA + trfA* with CPT (2 µg/mL) and MER (32 µg/mL) and SA5007 and SA3957*∆trfA* with CPT (16 µg/mL) and MER (8 µg/mL). Significant differences (*P* < 0.01; ANOVA) are indicated with an asterisk (*). (**B**) The sensitivity of strains to ROS was assessed by growing overnight cultures in the absence (left panel) or presence (right panel) of 0.75 mM H2O2 for 4 h, then measuring cell survival after serial dilutions and plating on TSA, showing altered sensitivities to ROS across different strains.

Spx orchestrates cellular responses to reactive oxygen species (ROS), including O₂⁻, H₂O₂, and OH·, thereby maintaining redox balance and protecting against oxidative stress (24). To examine more thoroughly the potential connection between dysfunctional Spx-mediated oxidative stress response and high levels of ceftaroline resistance, we quantified ROS production under various conditions using dihydrorhodamine (DHR) fluorescence as an indicator. Our results revealed a substantial increase in ROS production in SA5007 and SA3957*∆trfA* strains following exposure to meropenem, ceftaroline, and H_2_O_2_ (control), with meropenem eliciting the highest levels. Conversely, SA3957 and complemented SA3957*∆trfA+∆trfA* exhibited no significant changes in ROS production under any condition (Fig. 5A). Consistently, ceftaroline-resistant SA5007 and SA3957*∆trfA* strains were more susceptible to killing by H_2_O_2_, in line with a deficient Spx function, whereas ceftaroline-susceptible SA3957 and SA3957*∆trfA+trfA* strains behaved similarly to the control N315 strain (Fig. 5B). Together, these findings suggest that high-level ceftaroline resistance (SA5007 and SA3957*∆trfA*) involves impairment of the oxidative stress response with a defective Spx/TrfA pathway.

### Inactivation of *trfA* is associated with ClpX, PBP2a- and PBP1-mutation-mediated ceftaroline resistance

Through whole-genome sequencing (WGS), we identified that high level of ceftaroline resistance involved mutations in the *pbp1 (or pbpA*) gene (Tables 1 and 2). Subsequent analyses performed in *ΔtrfA*-mutant strains (Table 6) revealed identical *pbp1* (H499R), *mecA* (Y446N; E447K), and *clpX* (I418T) mutations at the same loci to those found in high-level CPT-resistant strains and in strains selected in the presence of meropenem before exposure to ceftaroline (6). The consistent association of TrfA with these mutations suggests its involvement in high-level CPT resistance and cell wall synthesis.

**TABLE 6:** Inactivation of *trfA* is associated with ClpX-, PBP2a-, and PBP1-mutation-mediated ceftaroline resistance^*a*^

Gene	SEA3*ΔtrfA*	S93*ΔtrfA*	S149*ΔtrfA*	AA change
*gyrB*	C > T	C > T	C > T	A419V
*gyrA*	C > T	C > T	C > T	S84L
*mecA*	C > T/ A > G	C > T/ A > G	C > T/ A > G	E447K/ Y446H
*capB*	T > C	T > C	T > C	C81R
*capD*	T > C	T > C	T > C	G599D
*atl*	T > C	T > C	T > C	Q637R
*mutS2*	A > T	A > T	A > T	V252I
*pbp1*	A > G	A > G	A > G	H499R
*pbp2*	C > T	C > T	C > T	S707L
*clpX*	G > A	G > A	G > A	T418I
*acC*	T > ins T	T > ins T	T > ins T	A277fs
*icaB*	T > C	T > C	T > C	F178L

^*a*^ Whole-genome sequencing (WGS) analysis of mutations in high-level ceftaroline resistance *trfA*-null mutant S3, S93, and SEA149 strains revealing identical *pbp1* (H499R), *mecA* (Y446N; E447K), and *clpX* (I418T) mutations at the same loci to those found in high-level CPT-resistant SA5007 strain and those selected in the presence of meropenem before exposure to ceftaroline.

### TrfA crosstalks to the second messenger cyclic di-3,5-adenosine monophosphate (c-di-AMP) signaling pathway

**Fig 6:**
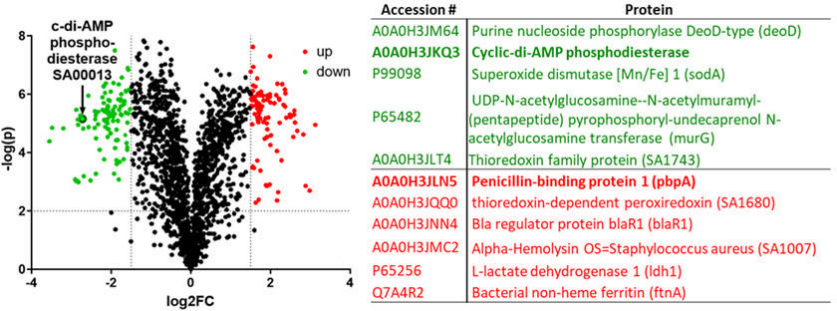
Proteomic analysis of differentially expressed proteins comparing SA5007 vs SA3957 strains exposed to ceftaroline (8 µg/mL and 2 µg/mL, respectively). Volcano plots depicting the up- (red dots) and downregulated (green dots) proteins. Relevant proteins are shown in the adjacent table.

To further understand the role of the Spx/TrfA pathway during ceftaroline resistance, differentially expressed proteins were identified in a comparative proteomic analysis. This analysis showed statistically significant changes, highlighted by red (upregulated) and green (downregulated) dots, based on thresholds of |log2 fold change| > 1 and *P* < 0.05 (Fig. 6). Among the proteins identified, the cyclic-di-AMP phosphodiesterase (SA0013) appeared significantly downregulated, suggesting altered nucleotide signaling. Other stress responses and metabolic proteins, including *sodA* and *murG*, were also downregulated. In contrast, proteins associated with virulence and oxidative stress defense, such as alpha-hemolysin (*SA1007*), ferritin (*bfrA*), and thioredoxin-dependent peroxidase (*SA1680*), were significantly upregulated.

**Fig 7:**
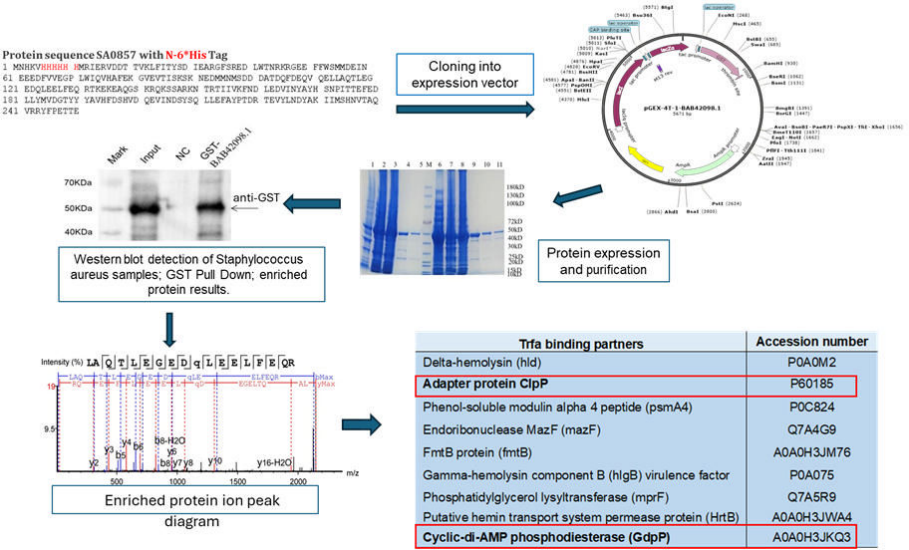
TrfA protein expression (bait) and GST pull-down–mass spectrometry assay. Following the steps shown in the figure including TrfA cloning, expression, purification, pull-down, and tandem affinity purification followed by mass spectrometry (TAP-MS) in the SA3957 strain, several partnering proteins cross-talking with TrfA were identified, including ClpP and c-di-AMP phosphodiesterase.

To investigate the physiological adaptations associated with high-level ceftaroline resistance, particularly the role of TrfA in cell wall modifications, we explored TrfA protein interactions using co-immunoprecipitation via tandem affinity purification followed by mass spectrometry (TAP-MS) in the SA3957 strain (Fig. 7). The Co-IP results confirmed a successful pulldown of TrfA and its associated protein complexes, providing insight into the molecular machinery underlying resistance. Mass spectrometry analysis on the immunoprecipitated complexes identified several TrfA binding partners, including cyclic-di-AMP phosphodiesterase (GdpP) (AO0HJ3KQ3), adapter protein ClpP (P60185), delta-hemolysin (Hld), gamma-hemolysin component B (HlgB), phenol-soluble modulin alpha 4 (Psmα4), ftnB (ferritin protein), and putative hemin transport and virulence-associated enzymes.

**Fig 8:**
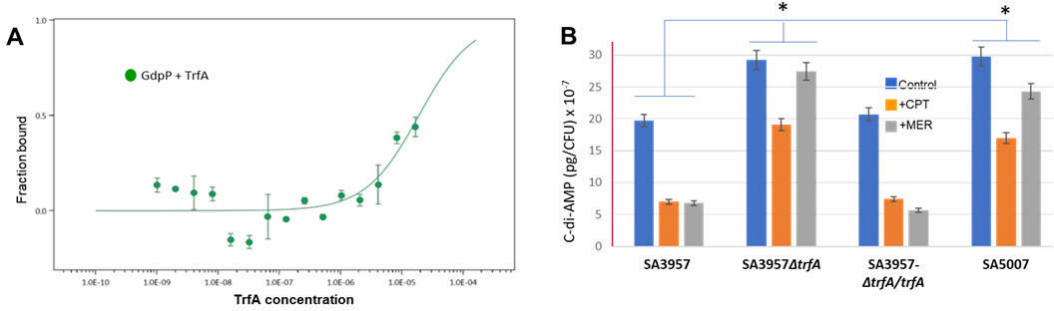
Analysis of TrfA/GdpP interactions. Determination of the normalized KD value for the binding between target protein GdpP and ligand TrfA based on MST (MicroScale Thermophoresis). (**A**) Levels of c-di-AMP determined by ELISA (Cayman Chemical) in cells treated +/- CPT (8 µg/mL) or MER (4 µg/mL). *Significantly different, *P* < 0.001 (ANOVA). (**B**) Levels of c-di-AMP determined by ELISA in cells treated +/- CPT (8 µg/mL) or MER (4 µg/mL). *Significantly different, *P* < 0.001 (ANOVA).

An interesting observation was that among the highest hits, we found that TrfA directly binds to GdpP, a phosphodiesterase that degrades cyclic di-3′,5′-adenosine monophosphate (c-di-AMP). C-di-AMP is a dinucleotide second messenger essential for growth and involved in various cellular processes, including cell size, cell osmolarity, and cell wall homeostasis (25, 26). Most importantly, GdpP has been identified as a critical potentiator of high-level β-lactam resistance (25, 27). To explore in more detail the TrfA/GdpP interactions, we used MicroScale Thermophoresis (Creative Proteomics, Shirley, NY) to determine binding affinities between both proteins (MST; Fig. 8A). This technology measures protein interactions by detecting the movement of fluorescent molecules through a temperature gradient. Briefly, the concentration of the fluorescently labeled GdpP molecule was kept constant at 10–150 nM, while TrfA (SA0857) was subjected to a twofold serial dilution, followed by the target protein labeling and binding affinity. The affinity analysis between both proteins showed strong concentration dependence, indicating specific binding (Fig. 8A). The affinity value determined was KD = 18.9 µM, indicating binding affinity and confirming specific binding interactions between TrfA and GdpP, which, functionally, may facilitate rapid cellular responses under antibiotic-induced stress conditions, reinforcing the role of the TrfA/c-di-AMP axis in resistance maintenance. Importantly, analysis of c-di-AMP levels showed increased basal levels in ceftaroline-resistant SA5007 and SA3957∆*trfA* strains compared with the corresponding ceftaroline-susceptible SA3957 and trans-complemented SA3957∆*trfA + trfA* strains, both at basal level and under exposure to ceftaroline and meropenem (Fig. 8B).

Thus, the present results showing TrfA/GdpP interaction and increased c-di-AMP suggest a mechanistic link between TrfA activity and altered c-di-AMP concentrations, which may be contributing to high-level ceftaroline resistance. Moreover, they suggest that these adaptations may compensate for the impaired function of PBP1, highlighting the bacteria’s ability to use alternative mechanisms to survive antibiotic stress.

## DISCUSSION

This study establishes, for the first time, a comprehensive model of high-level ceftaroline resistance in MRSA clinical strains, a significant advancement given ceftaroline’s role as a last-resort anti-MRSA agent. The findings underscore an emerging theme in antibiotic resistance: that collateral resistance can arise from prior exposure to unrelated β-lactams, in this case, carbapenems. This has important clinical implications, suggesting that prior carbapenem therapy may inadvertently prime MRSA for high-level ceftaroline resistance, thereby narrowing effective therapeutic options. Rather than arising from a single genetic alteration, the resistance phenotype uncovered here is multifactorial, involving the convergence of transcriptional rewiring, altered cell wall enzymatic function, and disruption of intracellular signaling. First, our results indicate that *rpoB* mutations serve not only as markers of rifampicin resistance but also as potent modulators of transcriptional responses, contributing to the evolution of high-level ceftaroline resistance. Whole-genome sequencing and serial passaging experiments demonstrated that only MRSA strains harboring non-synonymous *rpoB* mutations developed high-level ceftaroline resistance (MIC ≥32 µg/mL). None of the identified mutations were in the regions of binding of rifampicin (28, 29), which coincided with rifampicin sensitivity of the strains. These mutations, previously associated with altered RNA polymerase function and global transcriptional changes, may activate cryptic metabolic pathways and resistance determinants, as shown in other studies (30–32). In contrast, strains lacking *rpoB* mutations or containing only synonymous variants failed to achieve such resistance levels, underscoring the potentiating role of these mutations.

It has been shown that the *rpoB*-H481Y mutation in *S. aureus* leads to widespread transcriptomic changes compared to its isogenic parent, helping to explain altered virulence, persistence, and capsule production in clinical MRSA strains. The *rpoB*-A477D mutation has also been linked to pleiotropic effects, including altered levels of the Spx regulator (29). Consistent with these findings, strains in our study that were pre-exposed to meropenem and carried specific *rpoB* mutations—H481N, S464P, and Q468L—exhibited high-level ceftaroline resistance. Considering the proximity of the mutations found on this study with A477D *rpoB* location, it suggests that *rpoB* mutations may contribute to ceftaroline resistance and promote survival under conditions where Spx function is compromised.

The essential role of PBP1 as a transpeptidase in *S. aureus* cell division is well established (13, 14). We identified a key H499R substitution in the transpeptidase domain of PBP1 in both high-level ceftaroline-resistant clinical isolates and laboratory-evolved strains. This mutation likely not only impairs antibiotic binding but also alters regulatory feedback loops that enable persistent cell wall synthesis under antibiotic pressure. Consistent with this, previous studies have highlighted PBP1’s central role in cell division and septation in *S. aureus* (33, 34). Additionally, in *E. coli*, DNA replication and cell division have been shown to be tightly coupled and synchronized processes (35), suggesting that perturbation of one component may reverberate across essential cellular pathways. Building on our prior findings, we previously demonstrated that oxacillin-induced stress not only affects cell growth but also DNA replication rates, and that inhibition of PBP1 by β-lactams triggers the RecA-dependent SOS response, thereby increasing mutation rates and selecting for highly resistant MRSA strains (35).

At the center of this regulatory network lies the TrfA/Spx stress response pathway, a node that appears to coordinate oxidative stress adaptation and antibiotic survival. The basal overexpression of Spx in resistant strains and the lack of inducibility suggest a dysregulated state that may allow cells to bypass typical checkpoints governing cell wall integrity and redox balance. Such a bypass could facilitate the selection and persistence of *pbp1* and *mecA* variants during ceftaroline exposure. Moreover, the interaction between TrfA and GdpP uncovers a previously unappreciated crosstalk between protein degradation systems and nucleotide signaling. Elevated c-di-AMP in high-level ceftaroline-resistant clinical strains and in generated ceftaroline *trfA* mutants reflects either a loss of GdpP activity or altered TrfA-mediated regulation. Because c-di-AMP controls critical processes such as peptidoglycan synthesis and ion homeostasis, its accumulation may act as a buffering signal compensating for impaired PBP1 function. This mechanism could shift our understanding of how MRSA coordinates its internal circuitry in response to external antibiotic pressure. Reinforcing these findings, recently, Foster’s group has revealed across various MRSA strains (including COL, MRSA252, Mu50, USA300, and laboratory-derived MRSA constructs) that high-level resistance to methicillin is linked with a novel mode of cell division in the presence of antibiotics without the cell wall ring architecture (36). They demonstrated that the cell wall rings formed during division are due to PBP1 activity, which can be compensated for by *rpoB* mutations (36). It was also demonstrated that the transpeptidase activity of PBP1 is essential; its deficiency leads to thickened, abnormal septa, a phenotype mimicked by treatment with the PBP1-specific β-lactam meropenem (14).

What emerges is a broader framework for understanding resistance that transcends the classical view of single target mutations. Instead, our data suggests that sustained antibiotic exposure—particularly in the context of prior meropenem treatment—can select for multilayered regulatory rewiring. This includes *rpoB* mutations that modulate global transcription, cell wall remodeling through PBP1 substitutions, and signal transduction shifts via TrfA-GdpP-c-di-AMP interactions. Together, these findings demonstrate that resistance is not merely an outcome of evolutionary drift but rather a coordinated adaptive process involving stress pathways, proteostasis, and second messengers.

**Fig 9:**
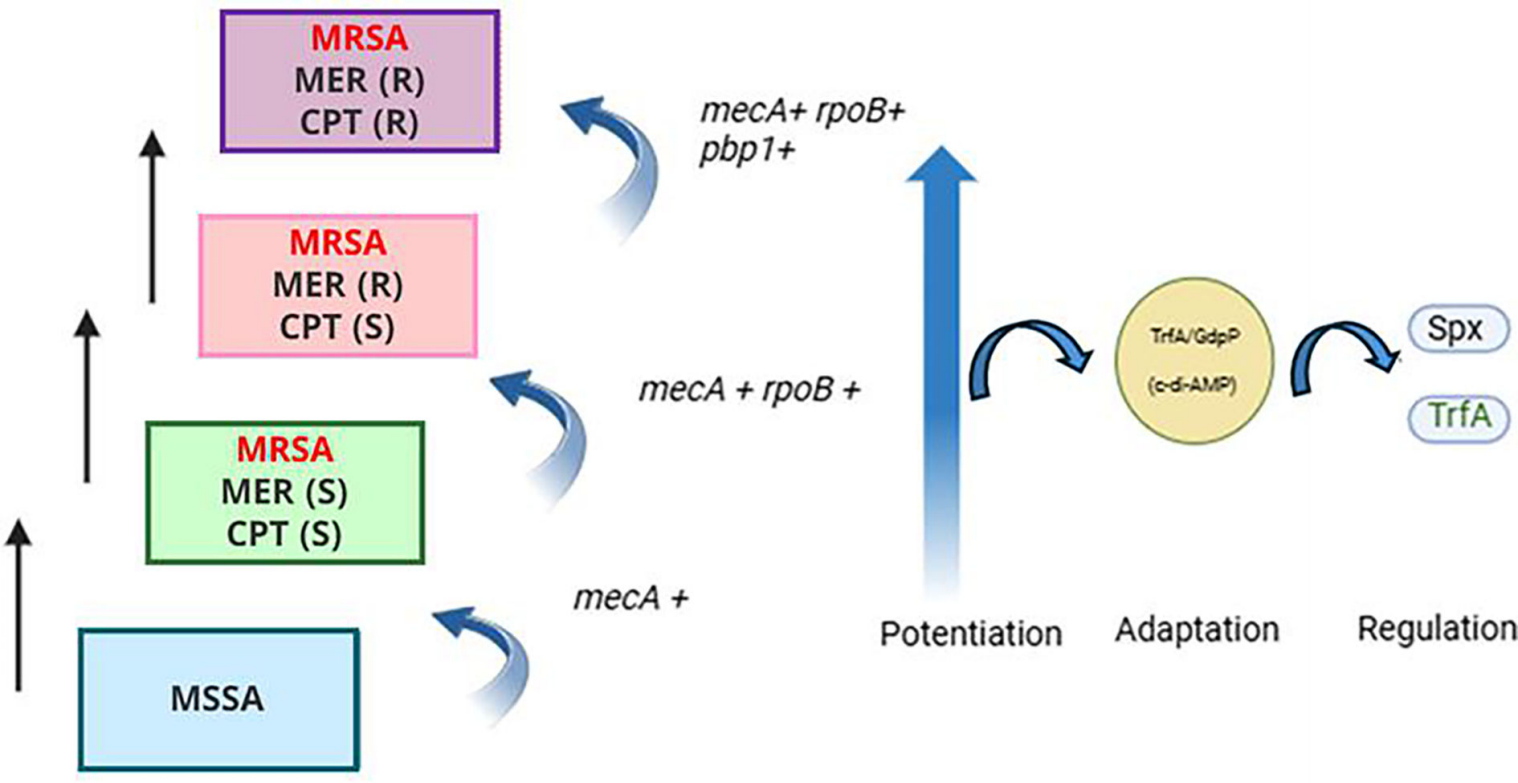
Model of acquisition of high-level CPT resistance in MRSA clinical strains. The present study defines a novel mechanistic framework for HIGH-level ceftaroline resistance beyond *mecA* mutations, in which RpoB plays a key link between carbapenems exposure and ceftaroline resistance emergence. In agreement with recent studies (36), *rpoB* alleles can act as “potentiator” mutations, allowing MRSA to adopt an alternative mode of cell division that bypasses the otherwise essential PBP1. Ceftaroline resistance is sustained through regulatory adaptations, where the Spx/TrfA oxidative stress response and c-di-AMP nucleotide signaling pathway compensate for impaired PBP1 function, ensuring long-term survival under ceftaroline pressure.

From a therapeutic standpoint, these insights advocate for re-evaluating sequential β-lactam use in clinical settings, especially among MRSA-infected patients. The model proposed here lays the foundation for these efforts and offers a new lens through which ceftaroline resistance can be predicted and ultimately mitigated. Taken together, our results support a model in which *rpoB* mutations potentiate resistance evolution, while PBP1 H499R and *mecA* variants sustain cell wall synthesis under drug pressure (Fig. 9). Disruption of the Spx/TrfA axis leads to redox imbalance and altered transcriptional responses, promoting further resistance. TrfA interaction with GdpP reveals a novel interface between stress adaptation and nucleotide signaling, wherein elevated c-di-AMP compensates for impaired PBP1 function and facilitates survival under high-level ceftaroline exposure. These insights point to potential therapeutic strategies targeting the TrfA/Spx/GdpP network to combat persistent MRSA infections.

## MATERIALS AND METHODS

### Bacterial strains and antimicrobial susceptibility testing

The clinical MRSA strains used in this study, along with the generated *trfA* mutants, are listed in Table 4. Antimicrobial susceptibility testing was performed in accordance with Clinical and Laboratory Standards Institute (CLSI) guidelines (33, 34). *Staphylococcus aureus* strains were subcultured and maintained on Trypticase Soy Agar with 5% sheep blood (Becton, Dickinson and Company, Sparks, MD), Mueller-Hinton (MH) agar (BBL Microbiology Systems, Cockeysville, MD), Trypticase Soy Agar (BBL Microbiology Systems), and LB broth (Difco, BD Biosciences), supplemented with appropriate antibiotics as needed (Sigma, St. Louis, MO; US Biochemicals, Cleveland, OH). Ceftaroline (CPT) and meropenem (MER) were obtained from Sigma-Aldrich (St. Louis, MO). Minimal inhibitory concentrations (MICs) for CPT and MER were determined using E-test strips (bioMérieux, Marcy l’Etoile, France) and broth dilution methods, and results were interpreted following CLSI criteria. For CPT, susceptibility breakpoints were defined as ≤1 mg/L (susceptible), 2 mg/L (intermediate), and ≥4 mg/L (resistant). While no CLSI breakpoints exist for MER against *S. aureus*, we conservatively categorized strains based on the cefoxitin breakpoint (≤4 mg/L) to define meropenem-susceptible (MER-S) strains, following the approach proposed by Jones RN et al. (37), using carbapenems as a surrogate marker for ceftaroline activity. Susceptibility testing was also employed to assess strain phenotypes and confirm strain identity (e.g., absence of cross-contamination in microplates) during long-term passage experiments, alongside whole-genome sequencing analyses. Reference strains ATCC 25923 and ATCC 29213 were used as quality controls in all susceptibility tests in accordance with CLSI standards.

Evolution experiments were conducted using a representative set of MRSA clinical strains that were initially susceptible to meropenem (MER). Strains were first selected for meropenem resistance by plating on agar containing 10 µg/mL of MER. Once resistance was established, the MER-resistant strains were subjected to subsequent selection of ceftaroline (CPT). For the progressive selection protocol, microplates were prepared with twofold increasing sub-inhibitory concentrations of either MER or CPT (0.25, 0.5, 1, 2, 4, 8, 16, 32, 64, and 128  mg/L). Each well was inoculated with 1  ×  10⁵ CFU/mL of the respective clinical strain. After 24 h of incubation at 37°C, 50  µL from the most turbid well with the highest antibiotic concentration was transferred to a fresh microplate containing the same gradient of antibiotic concentrations. This daily passaging process was continued for 24 days. Phenotypic analyses to confirm the identity and characteristics of emerging mutants were performed on days 0, 7, 15, and 25. At the conclusion of the experiment, genotypic confirmation was conducted using whole-genome sequencing (WGS). The mutants initially selected with MER (10  mg/L) and subsequently exposed to progressive CPT concentrations were designated as MER/CPT mutants.

Genomic DNA from MRSA strains evolved in the presence of antibiotics was extracted from overnight cultures grown in Mueller-Hinton Broth at 37°C using the DNeasy Blood and Tissue Kit (Qiagen, Germantown, MD). DNA libraries will be prepared using the Microbial Isolate Sequencing Solution (NO-MISS) Rapid Barcoding Kit V14 (SQK-RBK114.24). Sequencing was performed using Nanopore technology (Oxford, UK), with a target depth of 10–30 million reads per sample. Sequences were aligned to the reference genome using DNAstar-Lasergene, and genome annotation was performed using the PATRIC annotation service (38).

The *rpoB* gene (3.791 kb) from *S. aureus* Wis7 (D471G, S486L *rpoB* mutations) was amplified with DreamTaq Green PCR Master Mix (Thermo Fisher, Waltham, MA) using primers *rpoB*-F and *rpoB*-R (Table 7). The PCR product was cloned into pCR 2.1-TOPO TA (Invitrogen, Thermo Fisher) and transformed into One Shot TOP10 *E. coli*, with selection on LB agar (25 µg/mL kanamycin, blue/white screening). The resulting construct (7.722 kb) was purified using the QIAPrep Spin Miniprep Kit (Qiagen).

**TABLE 7:** Bacterial strains, plasmids, and primers used in this study

Strains	Phenotype	Reference
SA3957	Ceftaroline susceptible; MIC: 2 µg/mL	Varela et al. (6)
SA5007	Ceftaroline resistant; MIC: 32 µg/mL	Varela et al. (6)
SEA94	Ceftaroline susceptible; MIC: 0.5 µg/mL	Varela et al. (6)
SEA149	Ceftaroline susceptible; MIC: 0.75 µg/mL	Varela et al. (6)
WIS 7	Ceftaroline susceptible; MIC: 0.5 µg/mL	This study
WIS 14	Ceftaroline susceptible; MIC: 0.5 µg/mL	This study
*S. aureus* N315	Ceftaroline susceptible; MIC: 1.0 µg/mL	Ohta et al. (39)
SA3∆*trfA::tetk*	Ceftaroline resistant; MIC: 16 µg/mL	This study
SA149∆*trfA::tetk*	Ceftaroline resistant; MIC: 32 µg/mL	This study
SA93*trfA::tetk*	Ceftaroline resistance; MIC: 16 µg/mL	This study
SA3957∆*trfA::tetk*	Ceftaroline resistant; MIC: 16 µg/mL	This study
SA3957∆*trfA + trfA (psk265*)	Ceftaroline susceptible; MIC: 1 µg /mL	This study
AR612	ISP4-2-1; *ΔtrfA*	Renzoni et al. (16)
Plasmids		
psk265	Chloramphenicol resistant	Plata et al. (35)
pCR 2.1-TOPO TA	Kanamycin and ampicillin resistance	Plata et al. (35)
Primers		
*pbpA*-F	CAACAACCAGAACGAGGAAAGATATATG	This study
*pbpA* -R	CCT TCCAAATTGAATTTGGACGC	This study
*pbpA* - probe	AGTAATAGATAAAAAGGCGAGTGCC	This study
*trfA* -F	GAATTATTGGCTCAAACACTTGAAGG	This study
*trfA*-R	TGA TTAACGATAGTTATAGTCAG	This study
*trfA*-probe	GTTTGACGATTTAGAGCAAGTTATC	This study
*spx*-F	GAGGAAAATCATATTCCTTATAC	This study
*spx*-R	CCAATTATGATTGACGAGAAACGC	This study
*spx*-probe	TCAAAAGTTTTCCAAGAATTAAATG	This study
*rpoB-*F	CATTCTGAGAAGTATAAAAGCTTGA	This study
*rpoB-*R	TTAATCAGTAACTTCTTTTTGTGTTTCAGGAGC	This study

The plasmid was linearized with BamHI-HF (NEB), dephosphorylated with Quick CIP (NEB, Ipswich, MA), and ligated with BamHI-HF–linearized pSK265 (3 kb) using T4 DNA Ligase (Thermo Fisher). Recombinants (10.722 kb) were transformed into TOP10 *E. coli* and selected on TSA (25 µg/mL kanamycin, 12.5 µg/mL chloramphenicol). Plasmids were purified and electroporated into *S. aureus* RN4220 and transduced into Wis7 strain by 80α phage-mediated transduction.

### Labeling of PBPs with Bocillin FL

Bocillin FL labeling of 20 µg of membrane proteins was performed using 100 µM Bocillin FL (Molecular Probes, Eugene, OR), with the proteins incubated for 30 min at 35°C. The reaction was stopped by adding 4 × SDS-PAGE sample buffer. The labeled membrane protein concentrations were determined by the Bradford protein assay. Fifteen micrograms were loaded on a 4–12% bis-Tris gel (Invitrogen, Thermo Fisher), and the proteins were detected using an Azure biosystem (Dublin, CA).

### Membrane protein extraction

For the isolation of membrane proteins, strains were grown in MHB until mid-exponential phase, and pellets were resuspended in 600 µL of phosphate-buffered saline (PBS). Bacterial cells were disrupted by adding glass beads and using a FastPrep cell disrupter (MP Biomedicals, Santa Ana, CA), and the lysate was centrifuged at 8,000 × *g* for 10 min at 4°C. The supernatant fraction was centrifuged for an additional 5 min at 8,000 × *g* at 4°C to remove the beads, and then, the supernatant was transferred to ultracentrifuge tubes and centrifuged at 45,000 rpm in a Thermo Sorvall WX Ultra series WX80 centrifuge (Thermo Fisher) for 1 h at 4°C. The membrane pellet was resuspended in PBS, and total membrane proteins were quantified by the Bradford protein assay (Thermo Fisher) and stored at −80°C.

### Western blotting

Proteins (15 µg) were separated on 4 to 12% bis-Tris gels and blotted onto nitrocellulose blotting membranes (Thermo Fisher). The membranes were blocked using 5% low-fat milk in PBS. PBP2a was probed with monoclonal anti-PBP2a antibody (RayBiotech, Peachtree Corners, GA), and Spx and TrfA were produced by Thermo Fisher. Antibodies were used at a 1:2,000 dilution, followed by incubation with a secondary alkaline phosphatase-labeled goat anti-rabbit IgG(H + L) antibody at a 1/5,000 dilution. The labeled protein signal was detected using an Azure Biosystems imager (Dublin, CA).

### Analysis of gene expression by RT-PCR

Total RNA was extracted using the RNeasy isolation kit (Qiagen). The concentration and integrity of the RNA samples were assessed by *A*_260_/*A*_280_ spectrophotometry and gel electrophoresis. RNA samples were cleaned and treated with DNase following the manufacturer’s recommendations to avoid potential DNA contamination. RNA was prepared from cells collected at exponential phase of growth of SA3957 and SA5007, both untreated and treated with CPT.

Real-time reverse transcription-PCR (RT-PCR) analysis for RNA samples was performed using a SensiMix SYBR one-step kit (Qantace/Bioline, Taunton, MA) according to the manufacturer’s protocol. The level of gene expression compared with that for a sample considered the reference (value = 1) was determined using log_2_−(ΔΔ*C_T_*), where *C_T_* represents the threshold cycle value. The change (*n*-fold) in the transcript level (Δ*C_T_*) was calculated using the following equations: Δ*C_T_* = *C_T_* for test DNA – *C_T_* for reference cDNA, ΔΔ*C_T_* = Δ*C_T_* for the target gene − Δ*C_T_* for 16S rRNA, and amount of target = 2^−ΔΔ^*^CT^*. The quantity of cDNA for each experimental gene was normalized to the quantity of 16S cDNA in each sample. The oligonucleotide primers and probes are shown in Table 4.

Genomic DNA from a representative set of MRSA strains evolved in the presence of antibiotics was extracted from overnight cultures grown in Mueller-Hinton Broth at 37°C using the DNeasy Blood and Tissue Kit (Qiagen, Hilden, Germany). DNA libraries were prepared using the Nextera XT DNA Library Preparation Kit (Illumina, San Diego, CA) and sequenced with 50-nucleotide reads on a HiSeq 2000 instrument. Sequencing reads from 15 *S*. *aureus* genomes were assembled using the Pathosystems Resource Integration Center (PATRIC) genome assembly service with the “Full SPAdes” pipeline, which incorporates BayesHammer for error correction and SPAdes for assembly. Genome annotation was performed using the PATRIC annotation service (38).

### Cyclic-di-AMP assay

Strains were cultured in MHB media at 37°C until an OD_600_ of 0.2–0.4 was reached. The cultures were then diluted to an OD of 0.003 in MH media and regrown with or without 4 µg/mL CPT and 4 µg/mL MER until an OD of 0.5–1.0 was achieved. The cultures (OD = 5× volume) were pelleted by centrifugation, and the cell pellets were resuspended in lysis buffer (20 mM Tris pH 7.4, 1% Triton X-100, 100 µg/mL lysozyme, 5 U/mL DNase I), then incubated at room temperature for 20 min. Lysates were centrifuged at 15,000 × *g* for 5 min at 4°C. The supernatants were used for a c-di-AMP ELISA assay (Cayman Chemicals, Ann Arbor, MI) according to the manufacturer’s instructions. This assay quantifies cellular c-di-AMP by competitive binding to HRP-tagged c-di-AMP. Cyclic-di-AMP levels were calculated from the standard curve, normalized to culture OD, and presented as moles per OD.

### Detection of reactive oxygen species (ROS)

ROS production was measured using dihydrorhodamine 123 (DHR123) (Sigma-Aldrich, Burlington, MA) at a concentration of 0.9 µg/mL during exposure of SA3957 and SA3957*∆trfA + trfA* to CPT (2 µg/mL) and MER (32 µg/mL), and of SA5007 and SA3957*∆trfA* to CPT (16 µg/mL) and MER (8 µg/mL). ROS levels were quantified in cells treated with DHR123, with fluorescence normalized to cell density (OD_600_). Hydrogen peroxide (H2O2; 1 mM) was used as a positive control. To assess strain sensitivity to ROS, overnight cultures grew in the presence of 0.75 mM H2O2 for 4 h, followed by cell survival measurement through serial dilutions and plating on TSA, revealing varying sensitivities to ROS across strains. DHR123 becomes fluorescent upon oxidation to rhodamine 123 in the presence of intracellular ROS. Statistical significance was determined using ANOVA (*P* < 0.01).

### LC-MS/MS Analysis

#### Protein extraction

The pellet of untreated and treated cells with CPT, SA3957 (2 µg/mL) and SA5007 (8 µg/mL), was resuspended in urea-containing lysis solution and centrifuged at 14,600 × *g* for 15 min at 4°C. The supernatant was transferred to a new tube, and the pellet was discarded. Protein concentration was determined using the modified Bradford assay.

#### Tryptic digestion

Protein samples (20 µg) were treated with 1 mM dithiothreitol (DTT) for 1 h at room temperature to reduce disulfide bonds, followed by alkylation with 50 mM iodoacetamide (IAA) for 30 min. Trypsin (1:50) was added and incubated overnight at room temperature. The digestion was stopped by adding 1% trifluoroacetic acid (TFA). Peptides were desalted using C18 tips and dried using a SpeedVac at 38°C. Samples were reconstituted in 0.1% formic acid (FA) and 2% acetonitrile (ACN) for analysis. Proteomic profiling was performed using a TimsTOF mass spectrometer, Nano Elute UHPLC, and CaptiveSpray ion source (Bruker, Germany). A 4 µL peptide sample (4 µg) was injected and separated on a FIFTEEN C18 column (15 cm × 75 µm, 1.9 µm) with a 140 min run time. Solvent A was 0.1% FA in water, and solvent B was 0.1% FA in ACN. The gradient started at 5% B for 5 min, increased to 35% B over 115 min, then ramped to 95% B for 5 min, held for 10 min, and re-equilibrated at 5% B for 5 min. The flow rate was 300 nL/min, drying gas was 3 L/min at 150°C, and the capillary voltage was + 1.6 kV. The scan range was 150–2,200 m/z, and the instrument was operated in auto-MS/MS mode with CID fragmentation. Collision energy varied by precursor m/z from 23 eV to 65 eV. A fixed cycle time of 3 s was used, with a minimum intensity threshold of 500 counts per thousand. Samples were run in triplicates for each extraction method.

### MicroScale thermophoresis

The genes for *trfA* and *gdpP* were synthesized and cloned into the pCZN1 vector, and sequencing and PCR confirmed the correct sequence and quality of the constructs. These plasmids were then transformed into *E. coli* strains BL21(DE3) and Arctic-Express, and protein expression was induced by IPTG at different temperatures (15°C for *trfA* and 37°C for *gdpP*) to optimize yield. The proteins were extracted and purified using Ni-NTA affinity chromatography. Fusion proteins were eluted using a histidine-specific buffer, followed by SDS-PAGE analysis to confirm the successful purification and Western blot analysis to verify the identities of the proteins. The purified proteins, *trfA* (29.73 kDa) and *gdpP* (75.77 kDa), were obtained with a purity greater than 85%. The binding affinity between the target protein GdpP and ligand SA0857 (TrfA) was measured using MicroScale Thermophoresis. The target protein was labeled with a fluorescent dye, and serial dilutions of the ligand were prepared. In the MST assay, the labeled protein was kept at a constant concentration, while the ligand was diluted in a twofold series. Binding was assessed by mixing the target protein with the ligand and analyzing the samples in capillaries using the Monolith NT.115 instrument. A pretest ensured proper labeling and protein quality.
